# Morphological Study of Root Canals of Maxillary Molars by Cone-Beam Computed Tomography

**DOI:** 10.1155/2022/4766305

**Published:** 2022-01-18

**Authors:** Jackeline Magalhães, Christianne Velozo, Diana Albuquerque, Caio Soares, Hugo Oliveira, Maria Luíza Pontual, Flávia Ramos-Perez, Andrea Pontual

**Affiliations:** ^1^Department of Clinical and Preventive Dentistry, Federal University of Pernambuco, Recife, PE, Brazil; ^2^Department of Restorative Dentistry and Endodontics, Dental College of Pernambuco, University of Pernambuco, Recife, PE, Brazil

## Abstract

The aim of this study was to evaluate the root canal morphology of permanent maxillary molars by cone-beam computed tomography (CBCT) using the classifications of Weine et al. and Vertucci and to correlate the findings with sex, age, position in the dental arch, and prevalence of a second canal in the mesiobuccal root (MB2). A total of 414 scans were evaluated, corresponding to 1,000 teeth. The assessment consisted of coronal, axial, and sagittal reconstructions using i-CAT Workstation®. Type 0 was assigned when neither classification could be applied. The data were entered into an Excel spreadsheet and analyzed using SPSS. The chi-squared test or Fisher's exact test was used to compare the distribution of root canal morphology classified using the two systems. Analysis of the distribution of Weine types showed a predominance of type III in mesiobuccal roots, while type 0 predominated in distobuccal and palatal roots. Vertucci type IV predominated in mesiobuccal and distobuccal roots and type VII in palatal roots. There was no difference in the distribution of morphological canal types in permanent maxillary molars evaluated by CBCT according to sex, age group, or position in the dental arch of the patients. MB2 canals were identified in 68.4% of the teeth evaluated.

## 1. Introduction

The main objective of endodontic treatment is the cleaning, disinfection, and three-dimensional filling of the root canal system in order to prevent reinfection and to cure the infection [1]. Improvements in endodontic treatment have been achieved over the past decades by the development of new materials, techniques, and devices in order to increase success rates [2]. However, the success of this treatment depends on accurate knowledge about the morphology of root canals, which is very complex due to the splitting and union of canals during their trajectory [3].

Root canal morphology shows distinct configurations between different populations and between different tooth groups, particularly among molars [3]. The maxillary molars have complex anatomy, and their root canal system is characterized by wide variations, a fact that represents a constant challenge for the dentist [4]. These teeth generally have three roots (mesiobuccal (MB), distobuccal (DB), and palatal (P)) and can contain up to three mesial canals, two distal canals, and two palatal canals [5, 6]. In addition, the MB root can contain a second root canal system (MB2) that can range from a single to multiple canals [7].

The main causes of endodontic treatment failure include incomplete fillings and the presence of untreated root canals and thus result in the persistence of microorganisms in intact areas that become inaccessible and incompletely filled, leading to the failure of endodontic treatment [2, 8, 9]. The lack of knowledge of root canal morphology can compromise the identification of additional canals and contribute to this failure. Knowledge of the anatomical variations of root canals is therefore essential in order to avoid undesirable failures and to increase the chances of successful endodontic treatment [3, 10].

Periapical radiographs continue to be the most widely used images for root canal assessment during endodontic treatment that can provide useful information for the clinician. Despite their widespread use, these images provide limited information about morphological variations, neighboring bone densities, X-ray angulations and contrast, and factors that can influence radiographic interpretation [11]. The absence of three-dimensional information and the presence of areas of interest masked by overlapping structures in the images interfere with the establishment of the correct diagnosis [12].

In endodontics, cone-beam computed tomography (CBCT) has been shown to be very useful in assessing the morphology and location of the root canal, as well as in the visualization of the root's anatomy, differential diagnosis between endodontic and nonendodontic pathologies, evaluation of alveolar and root fractures, analysis of internal and external resorption, presurgical endodontic planning, evaluation of root preparation, obturation, retreatment, detection of bone lesions, and endodontic research [11]. Since CBCT provides an in-depth view of the morphology of the root canal, this technique may be indicated to obtain information on complex anatomies [13].

The aim of the present study was to evaluate the root canal morphology of permanent maxillary molars by CBCT and its correlation with sex, age, position (right/left) in the dental arch, and the prevalence of MB2.

## 2. Materials and Methods

### 2.1. Ethical Aspects

The study was approved by the Ethics Committee of the Federal University of Pernambuco (approval number 2.224.057) and was conducted in accordance with the ethical guidelines of Resolution 466/12 of the National Health Council that regulates research involving humans.

### 2.2. Sample

The study was conducted at the Dental Radiology Clinic, Federal University of Pernambuco, Recife, Pernambuco, Brazil, in a dark room to optimize visualization of the root canal anatomy.

All maxillary CBCT scans of patients seen between January 2016 and January 2017 at a private dental radiology service were retrieved. Only scans that met the predefined inclusion and exclusion criteria were selected for the sample of the present study.

### 2.3. Cone-Beam Computed Tomography

The scans were acquired with the i-CAT Next Generation^®^ CBCT scanner (Imaging Sciences International, Hatfield, Pennsylvania, USA) that provides volumetric data within a few seconds. This device uses a fixed voltage and current of 120 kVp and 5.5 mA, respectively, and a focal spot size of 0.5 mm. The digital image detector is composed of a 20 × 25 cm flat amorphous silicon plate. For this study, the image was captured with 14 bits of resolution and a voxel pixel of 0.3/0.3 by the same operator. The image was acquired in a single 360-degree rotation around the patient. The scan volume is 17 cm in diameter by 13 cm in height.

For acquisition of the CBCT images, the patient was positioned in the device in such a way that the occlusal plane remained parallel to the ground, following the image acquisition protocol recommended by the manufacturer. After the acquisition, the images appear on the computer screen within a few seconds and are stored in xstd format (xoran extension) for subsequent evaluation.

### 2.4. Inclusion and Exclusion Criteria

Scans of patients ranging in age from 10 to 90 years with permanent maxillary first and/or second molars were included in the study.

CBCT scans of the maxilla showing maxillary first and/or second molars with endodontic treatment, extensive caries, incomplete rhizogenesis, fused roots, root remnants, and image artifacts resulting from dental implants in adjacent teeth were excluded. CBCT scans of the maxilla without adequate diagnostic quality and scans of the mandible were also not included in the study.

### 2.5. Calibration

The tomographic images were evaluated by an examiner who had been previously trained and calibrated in the proposed assessment using this imaging modality. Calibration consisted of assessing tomographic images from 30 teeth (maxillary first and second molars), selected according to the predefined inclusion and exclusion criteria, under the same established conditions. These teeth were evaluated and reevaluated after one week to determine intraexaminer agreement for the morphological types attributed to the root canals according to the classifications of Weine et al. [14] and Vertucci [15]. The data obtained were entered into an Excel^®^ spreadsheet (Microsoft Corporation, Redmond, Washington, USA) and then transferred to the SPSS software (Statistical Package for the Social Sciences® version 20). Intraexaminer reliability was evaluated using Cohen's kappa test. Calibration was performed, and a satisfactory kappa index was obtained (good to perfect classification).

### 2.6. Selection of Sample

A total of 840 CBCT scans from the database performed for different reasons were examined. Of these, 426 scans were not included in the assessment because of (1) insufficient field of view, (2) presence of at least one of the exclusion criteria attributed to all maxillary molars, and (3) poor image quality for diagnostic purposes. Among all maxillary scans examined, 1,350 teeth were excluded from the sample because they met one or more of the predefined exclusion criteria. The sample consisted of 414 maxillary scans, including 238 female patients and 176 male patients ranging in age from 10 to 89 years. A total of 1,000 teeth were evaluated: 505 on the right side and 495 on the left side (Table 1).

### 2.7. Evaluation of the CBCT Images

The root canal morphology of the molars was evaluated in a dark and silent room at the Dental Radiology Clinic of UFPE using a computer with a 22″ monitor and i-CAT Workstation^®^ (Imaging Sciences International, Pennsylvania, USA). The interface of the program was used to evaluate the root canals in coronal, axial, and sagittal sections.

In the window of axial sections, the examiner determined the section where the long axis of the tooth, first or second molar, had the largest diameter, visualized in its long axis, and the examiner determined the central sagittal section. Through this window of sections, the volume was rotated in order to align the long axis of the tooth with the vertical plane.

**Figure 1:**
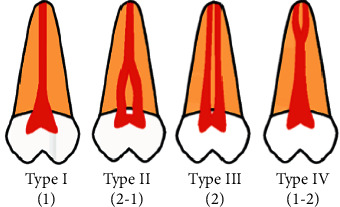
Illustrative image of the morphological classification of root canals according to Weine et al. [14].

**Figure 2:**
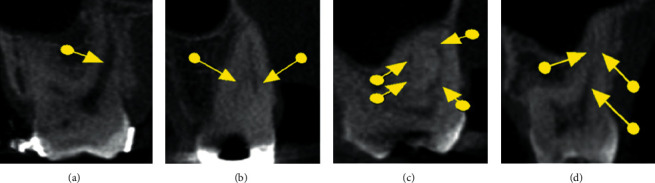
Coronal sections showing the different morphological types proposed by Weine et al. [14], (a) type I, (b) type II, (c) type III, and (d) type IV.

**Figure 3:**
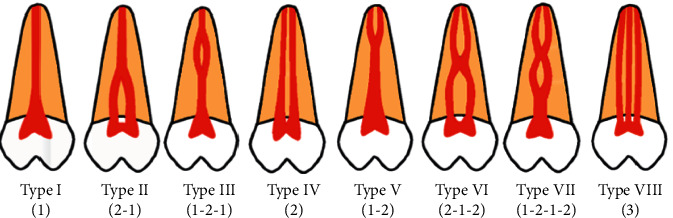
Illustrative image of the morphological classification of root canals according to Vertucci [15].

**Figure 4:**
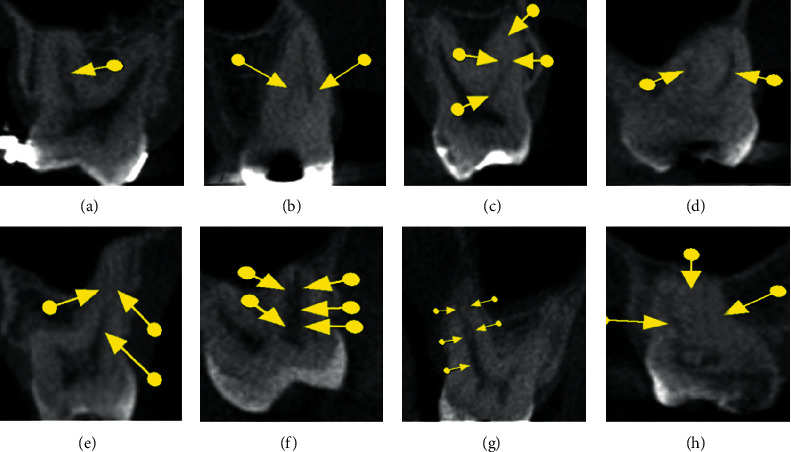
Coronal sections showing the different morphological types proposed by Vertucci [15]: (a) type I, (b) type II, (c) type III, (d) type IV, (e) type V, (f) type VI, (g) type VII, and (h) type VIII.

Prior to the assessment, the images were processed using the same filter (sharpness, brightness, and contrast) in order to enable better visualization and standardization of the reconstructions. Next, the morphology of the root canals was classified according to Weine et al. [14] and Vertucci [15] (Table 2 and Figures 1–4), the classifications most commonly used in the literature. In addition to the classification of root canal morphology, the presence of MB2 canals in the maxillary molars was evaluated. For statistical purposes, type 0 was assigned to cases that did not meet the criteria of either classification.

### 2.8. Data Analysis

The data of the tomographic assessments were entered into an Excel^®^ spreadsheet (Microsoft Corporation, Redmond, Washington, USA), including patient age and sex and tooth and type of internal root canal configuration according to the two anatomical classifications used, for subsequent statistical analysis. A database was built in SPSS 20 (Statistical Package for the Social Sciences) for analysis of the data. The chi-squared test for homogeneity was applied to compare the distribution of root canal morphology classified according to Weine et al. [14] and Vertucci [15] among sexes, age groups, and sides of the dental arches of the patients evaluated. When the assumptions of the chi-squared test were not met, Fisher's exact test was applied. For statistical analysis, the following age categories were established: children and teenagers (0 to 17 years), adults (18 to 65 years), middle-aged adults (66 to 79 years), and older adults (80 to 99 years). A level of significance of 5% was adopted.

## 3. Results and Discussion

In the present study, type 0 was attributed to teeth whose root canal morphology could not be classified according to the criteria of Weine et al. [14].

The prevalence of the morphological canal types according to the classification of Weine et al. [14] and Vertucci [15] found in the total sample is shown in Table 3.

Table 4 shows the distribution of Weine canal types for MB, DB, and palatal roots, respectively, according to sex, age group, and dental arch of the patient evaluated.

Table 5 shows the distribution of Vertucci canal types for MB, DB, and palatal roots, respectively, according to sex, age group, and dental arch of the patient evaluated.

The distribution of MB2 canals in the sample studied according to sex, age group, and dental arch is shown in Table 6.

The present study showed marked anatomical variations in the root canal morphology of maxillary molars. Knowing that anatomical features influence the success of endodontic treatment, dentists must be aware of these anatomical complexities since inadequate knowledge can result in untreated canals and root canal perforations or transportation [16].

Using the classification proposed by Weine et al. [14], we observed a higher prevalence of canal morphology type III in MB roots of maxillary molars. Similar results have been reported by others CBCT studies [17, 18]. In contrast, another study [19] that evaluated root canals under a surgical microscope found Weine type I to be the most prevalent in MB roots, followed by type III.

The root canal morphology of 812 maxillary first and second molars in a Greek population by CBCT using the classification of Weine et al. [14] was evaluated, and type III was the most prevalent in MB roots [20], in agreement with the present results. However, in DB and P roots, the authors found a predominance of type I [20]. As observed in the present study, there were no significant differences in morphological canal types between sexes.

The anatomy of MB, DB, and P root canals of 442 maxillary molars in a Ugandan population using a clearing technique was analyzed, and the authors [21] found Weine type I to be the most prevalent morphology in all roots. These results differ from the present study in which type III predominated in MB roots and type 0 (root canals that could not be classified) in DB and P roots. The differences between the results reported by these authors and the present findings might be explained by the method used for the evaluation of root canal morphology. In addition, the divergences may also be attributed to the assessment criteria established, population differences, inclusion/exclusion criteria, or the small sample size when compared to the present study.

In the present study, using the classification proposed by Vertucci [15], the most prevalent morphological types found in MB, DB, and P roots were types IV, IV, and VII, respectively. In contrast, others authors obtained divergent results using cleared teeth and reported a higher prevalence of morphological types II, I, and I in MB, DB, and P roots, respectively [22].

In another study [23], types II, III, and IV were the most common morphological types in MB roots of maxillary first molars, and the first and second molars generally exhibited types I and II, in contrast to the present results. Furthermore, these authors found a case of morphological type VIII in an MB root, as also reported by a study [24]. Similar results were observed in the present study.

A retrospective study [25] evaluated the root canal morphology of maxillary first and second molars by CBCT in a Turkish population. Using the classification of Vertucci [15], the most common morphological types in MB roots were types I and II, followed by type IV. The root canal configuration of 60 maxillary first molars in the Khasi population of Meghalaya using canal staining and a clearing technique was investigated and obtained results similar [26] to those of the present study in which type IV was the most prevalent in the MB root.

The prevalence of MB2 canals in maxillary molars was high in the present study, irrespective of sex, age group, or side of the arch evaluated (*p* > 0.05). Aydin [16] evaluated 402 CBCT scans of the maxillary first and second molars in a Turkish population and detected MB2 canals in 79.34% and 53.14% of cases, respectively. In a CBCT study of the root canal morphology of maxillary molars in a Chinese subpopulation, MB2 canals were detected in the first molars of 68.3% of cases and in the second molars of 23.8%. The authors also found that when MB2 was present, Vertucci type IV was the most common morphology [27], in agreement with the results of the present study. MB2 canals in the maxillary first molar of 46% of cases and in the maxillary second molars of 14% were detected and the authors identified Vertucci type IV morphology as the most prevalent [28]. Authors evaluated 509 maxillary first and second molars by CBCT in a Chinese subpopulation and observed that when MB2 was present, type IV was the most common configuration in first molars, followed by types V and II [29]. The root canal morphology of maxillary first and second molars by CBCT in an Indian population was investigated, and morphological type IV was present in 50% and 38.6% of first and second molars with MB2 canals, respectively [30]. These results agree with the present study in which type IV was the most prevalent in MB roots, in addition to a high incidence of this fourth canal.

In another study [29], Vertucci [15] type I was the most prevalent morphological configuration in DB and P roots of maxillary molars. Bhuyan et al. [26] found type I, followed by types II and V, to be the most common morphology in DB roots and type I followed by type II in P roots. A total of 250 first and second molars extracted from an Iranian population by CBCT were evaluated and observed that Vertucci [15] type I was the most prevalent in DB and P roots [31]. These findings disagree with the results of the present study that showed greater morphological variations in these roots. Racial divergence may explain these variations.

In a case series in which five teeth, maxillary first and second molars, with bifurcated palatal canals were identified, the authors highlighted that the assumption of a single canal in all palatal roots of maxillary molars needs to be changed [32]. Within this context, a study [33] identified five cases of second molars with two or three canals in the palatal root. The findings of the present study corroborate the results of these authors since variations were observed in the morphology of the palatal canal, especially when the classification proposed by Vertucci [15] was used.

We found no significant differences in the prevalence of the different morphological canal types between sexes. Similar results have been reported by [22, 25, 34]. Regarding patient age, there were no significant differences between the predefined age groups [34].

Further studies investigating the root canal morphology of permanent maxillary molars are needed because of the complexity and different anatomical configurations of these teeth [3, 4] Knowledge of these configurations is essential to identify and treat additional canals and to increase the chances of successful endodontic treatment [4, 8, 10].

## 4. Conclusions

There were no differences in the distribution of the different morphological canal types in permanent maxillary molars evaluated by CBCT according to sex, predefined age group, or position in the dental arch of the patients. MB2 canals were identified in 68.4% of the teeth evaluated.

## Figures and Tables

**Table 1:** Scans excluded from the sample, maxillary teeth excluded and included from the sample

Scans excluded from the sample		Number
Scans of the mandible (insufficient FOV)		245
Scans without diagnostic quality		7
Scans of the maxilla in which all first and second molars met one or more exclusion criteria		174
Total		**426**
Exclusion criteria number		758
Absent		14
Extensive caries		15
Incomplete rhizogenesis		28
Deciduous molars		257
Endodontic treatment		80
Dental implant		14
Root remnant		49
Image artifact		54
Fused roots (MB, DB, and P)		60
Fused roots (MB and DB)		4
Fused roots (MB and P)		17
Fused roots (DB and P)		14
Total		**1,350**
Maxillary teeth included in the sample		
Sex (mean age, years)	Number of scans evaluated	Number of teeth evaluated
Male (47.22)	176	238
Female (47.85)	438	562
Total	**414**	**1,000**

MB: mesiobuccal; DB: distobuccal; P: palatal.

**Table 2:** Classification of the root canal system proposed by Weine et al. [14] and Vertucci [15].

Morphological type by Weine et al. [14]	Description
Type I	Single canal from the pulp chamber to the apex.
Type II	Two separate canals leave the pulp chamber and join short of the apex to form one canal.
Type III	Two separate and distinct canals from the pulp chamber to the apex.
Type IV	A single canal leaves the pulp chamber and divides into two canals with two separate foramina.
Morphological type by Vertucci [15]	Description
Type I	A single canal extending from the pulp chamber to the apex.
Type II	Two canals leave the pulp chamber and join short of the apex to form a single canal.
Type III	A single canal leaves the pulp chamber, divides into two inside the root, and then merges to form one canal.
Type IV	Two separate and distinct canals extending from the pulp chamber to the apex.
Type V	A single canal leaves the pulp chamber and divides into two canals with separate apical foramina.
Type VI	Two separate canals leave the pulp chamber, merge in the body of the root, and redivide short of the apex to exit as two distinct canals.
Type VII	A single canal leaves the pulp chamber, divides into two canals that then merge in the middle third of the root, and finally redivide into two distinct canals short of the apex.
Type VIII	Three separate canals extending from the pulp chamber to the apex.

**Table 3:** Prevalence of morphological root canal types according to the classifications proposed by Weine et al. [14] and Vertucci [15].

Classification	Mesiobuccal	Distobuccal	Palatal
	Prevalence (%)	Prevalence (%)	Prevalence (%)
Weine et al. [14]			
Type 0	304	602	605
	(30.4)	(60.2)	(60.5)

Type I	2	43	266
	(0.2)	(4.3)	(26.6)

Type II	9	4	17
	(0.9)	(0.4)	(1.7)

Type III	684	344	94
	(68.4)	(34.4)	(9.4)
Type IV	1	7	18
	(0.1)	(0.7)	(1.8)
Vertucci [15]			
Type I	2	43	266
	(0.2)	(4.3)	(26.6)

Type II	9	4	17
	(0.9)	(0.4)	(1.7)

Type III	12	50	160
	(1.2)	(5)	(16)

Type IV	684	345	92
	(68.4)	(34.5)	(9.2)

Type V	1	7	18
	(0.1)	(0.7)	(1.8)

Type VI	83	260	133
	(8.3)	(26)	(13.3)

Type VII	29	247	291
	(2.9)	(24.7)	(29.1)

Type VIII	180	44	23
	(18)	(4.4)	(2.3)

**Table 4:** Distribution of the different morphological types proposed by Weine et al. [14] for mesiobuccal roots, distobuccal roots, and palatal roots according to sex, age group, and dental arch of the patient evaluated.

Mesiobuccal roots according to sex, age group, and dental arch of the patient evaluated								
Weine classification	Sex		Age group				Arch	
	Male (*n* = 438)	Female (*n* = 562)	Children (*n* = 28)	Adults (*n* = 845)	Middle-aged (*n* = 114)	Older adults (*n* = 13)	Right (*n* = 505)	Left (*n* = 495)
0	121	183	5	263	33	3	169	135
	(27.6%)	(32.6%)	(17.9%)	(31.1%)	(28.9%)	(23.1%)	(33.5%)	(27.3%)

Type I	2	0	1	1	0	0	0	2
	(0.5%)	(0.0%)	(3.6%)	(0.1%)	(0.0%)	(0.0%)	(0.0%)	(0.4%)

Type II	4	5	1	8	0	0	4	5
	(0.9%)	(0.9%)	(3.6%)	(0.9%)	(0.0%)	(0.0%)	(0.8%)	(1.0%)

Type III	311	373	21	572	81	10	332	352
	(71.0%)	(66.4%)	(75.0%)	(67.7%)	(71.1%)	(76.9%)	(65.7%)	(71.1%)

Type IV	0	1	0	1	0	0	0	1
	(0.0%)	(0.2%)	(0.0%)	(0.1%)	(0.0%)	(0.%)	(0.0%)	(0.2%)

*p* value^1^	0.157		0.191				0.078	
Distobuccal roots according to sex, age group, and dental arch of the patient evaluated								
Weine classification	Sex		Age group				Arch	
	Male (*n* = 438)	Female (*n* = 562)	Children (*n* = 28)	Adults (*n* = 845)	Middle-aged (*n* = 114)	Older adults (*n* = 13)	Right (*n* = 505)	Left (*n* = 495)
0	262	340	16	518	61	7	297	305
	(59.8%)	(60.5%)	(57.1%)	(61.3%)	(53.5%)	(53.8%)	(58.8%)	(61.6%)

Type I	19	24	3	33	7	0	21	22
	(4.3%)	(4.3%)	(10.7%)	(3.9%)	(6.1%)	(0.0%)	(4.2%)	(4.4%)

Type II	2	2	0	3	1	0	3	1
	(0.5%)	(0.4%)	(0.0%)	(0.4%)	(0.9%)	(0.0%)	(0.6%)	(0.2%)

Type III	153	191	9	285	44	6	181	163
	(34.9%)	(34.0%)	(32.1%)	(33.7%)	(38.6%)	(46.2%)	(35.8%)	(32.9%)

Type IV	2	5	0	6	1	0	3	4
	(0.5%)	(0.9%)	(0.0%)	(0.7%)	(0.9%)	(0.0%)	(0.6%)	(0.8%)

*p* value^1^	0.951		0.503				0.735	
Palatal roots according to sex, age group, and dental arch of the patient evaluated								
Weine classification	Sex		Age group				Arch	
	Male	Female	Children	Adults	Middle-aged	Older adults	Right	Left
	(*n* = 438)	(*n* = 562)	(*n* = 28)	(*n* = 845)	(*n* = 114)	(*n* = 13)	(*n* = 505)	(*n* = 495)
0	266	339	17	510	69	9	306	299
	(60.7%)	(60.3%)	(60.7%)	(60.4%)	(60.5%)	(69.2%)	(60.6%)	(60.4%)

Type I	121	145	11	218	34	3	129	137
	(27.6%)	(25.8%)	(39.3%)	(25.8%)	(29.8%)	(23.1%)	(25.5%)	(27.7%)

Type II	8	9	0	16	1	0	7	10
	(1.8%)	(1.6%)	(0.0%)	(1.9%)	(0.9%)	(0.0%)	(1.4%)	(2.0%)

Type III	37	57	0	85	8	1	51	43
	(8.4%)	(10.1%)	(0.0%)	(10.1%)	(7.0%)	(7.7%)	(10.1%)	(8.7%)

Type IV	6	12	0	16	2	0	12	6
	(1.4%)	(2.1%)	(0.0%)	(1.9%)	(1.8%)	(0.0%)	(2.4%)	(1.2%)

*p* value^2^	0.746		-				0.488	

^1^Fisher's exact test; ^2^chi-squared test for homogeneity.

**Table 5:** Distribution of the different morphological types proposed by Vertucci [15] for mesiobuccal roots, distobuccal roots, and palatal roots according to sex, age group, and dental arch of the patient evaluated.

Mesiobuccal roots according to sex, age group, and dental arch of the patient evaluated	Sex		Age group				Arch	
Vertucci classification	Male (*n* = 438)	Female (*n* = 562)	Children (*n* = 28)	Adults (*n* = 845)	Middle-aged (*n* = 114)	Older adults (*n* = 13)	Right (*n* = 505)	Left (*n* = 495)
Type I	2	0	1	1	0	0	0	2
	(0.5%)	(0.0%)	(3.6%)	(0.1%)	(0.0%)	(0.0%)	(0.0%)	(0.4%)

Type II	4	5	1	8	0	0	4	5
	(0.9%)	(0.9%)	(3.6%)	(0.9%)	(0.0%)	(0.0%)	(0.8%)	(1.0%)

Type III	3	9	1	10	1	0	5	7
	(0.7%)	(1.6%)	(3.6%)	(1.2%)	(0.9%)	(0.0%)	(1.0%)	(1.4%)

Type IV	311	373	21	572	81	10	332	352
	(71.0%)	(66.4%)	(75.0%)	(67.7%)	(71.1%)	(76.9%)	(65.7%)	(71.1%)

Type V	0 (0.0%)	1 (0.2%)	0 (0.0%)	1 (0.1%)	0 (0.0%)	0 (0.0%)	0 (0.0%)	1 (0.2%)

Type VI	27 (6.2%)	56 (10.0%)	1 (3.6%)	73 (8.6%)	8 (7.0%)	1 (7.7%)	42 (8.3%)	41 (8.3%)

Type VII	10 (2.3%)	19 (3.4%)	2 (7.1%)	22 (2.6%)	4 (3.5%)	1 (7.7%)	15 (3.0%)	14 (2.8%)

Type VIII	81 (18.5%)	99 (17.6%)	1 (3.6%)	158 (18.7%)	20 (17.5%)	1 (7.7%)	107 (21.2%)	73 (14.7%)

*p* value^1^	0.114		-				0.146	
Distobuccal roots according to sex, age group, and dental arch of the patient evaluated								
Vertucci classification	Sex		Age group				Arch	
	Male (*n* = 438)	Female (*n* = 562)	Children (*n* = 28)	Adults (*n* = 845)	Middle-aged (*n* = 114)	Older adults (*n* = 13)	Right (*n* = 505)	Left (*n* = 495)
Type I	19	24	3	33	7	0	21	22
	(4.3%)	(4.3%)	(10.7%)	(3.9%)	(6.1%)	(0.0%)	(4.2%)	(4.4%)

Type II	2	2	0	3	1	0	3	1
	(0.5%)	(0.4%)	(0.0%)	(0.4%)	(0.9%)	(0.0%)	(0.6%)	(0.2%)

Type III	19	31	3	41	6	0	20	30
	(4.3%)	(5.5%)	(10.7%)	(4.9%)	(5.3%)	(0.0%)	(4.0%)	(6.1%)

Type IV	153	192	9	285	45	6	182	163
	(34.9%)	(34.2%)	(32.1%)	(33.7%)	(39.5%)	(46.2%)	(36.0%)	(32.9%)

Type V	2 (0.5%)	5 (0.9%)	0 (0.0%)	6 (0.7%)	1 (0.9%)	0 (0.0%)	3 (0.6%)	4 (0.8%)

Type VI	114 (26.0%)	146 (26.0%)	3 (10.7%)	227 (26.9%)	27 (23.7%)	3 (23.1%)	136 (26.9%)	124 (25.1%)

Type VII	112 (25.6%)	135 (24.0%)	10 (35.7%)	209 (24.7%)	24 (21.1%)	4 (30.8%)	122 (24.2%)	125 (25.3%)

Type VIII	17 (3.9%)	27 (4.8%)	0 (0.0%)	41 (4.9%)	3 (2.6%)	0 (0.0%)	18 (3.6%)	26 (5.3%)

*p* value^1^	0.956		-				0.531	
Palatal roots according to sex, age group, and dental arch of the patient evaluated								
Vertucci classification	Sex		Age group				Arch	
	Male (*n* = 438)	Female (*n* = 562)	Children (*n* = 28)	Adults (*n* = 845)	Middle-aged (*n* = 114)	Older adults (*n* = 13)	Right (*n* = 505)	Left (*n* = 495)
Type I	121	145	11	218	34	3	129	137
	(27.6%)	(25.8%)	(39.3%)	(25.8%)	(29.8%)	(23.1%)	(25.5%)	(27.7%)

Type II	8	9	0	16	1	0	7	10
	(1.8%)	(1.6%)	(0.0%)	(1.9%)	(0.9%)	(0.0%)	(1.4%)	(2.0%)

Type III	69	91	5	134	18	3	85	75
	(15.8%)	(16.2%)	(17.9%)	(15.9%)	(15.8%)	(23.1%)	(16.8%)	(15.2%)

Type IV	36	56	0	83	8	1	51	41
	(8.2%)	(10.0%)	(0.0%)	(9.8%)	(7.0%)	(7.7%)	(10.1%)	(8.3%)

Type V	6	12	0	16	2	0	12	6
	(1.4%)	(2.1%)	(0.0%)	(1.9%)	(1.8%)	(0.0%)	(2.4%)	(1.2%)

Type VI	56	77	4	111	15	3	58	75
	(12.8%)	(13.7%)	(14.3%)	(13.1%)	(13.2%)	(23.1%)	(11.5%)	(15.2%)

Type VII	132	159	8	245	35	3	150	141
	(30.1%)	(28.3%)	(28.6%)	(29.0%)	(30.7%)	(23.1%)	(29.7%)	(28.5%)

Type VIII	10	13	0	22	1	0	13	10
	(2.3%)	(2.3%)	(0.0%)	(2.6%)	(0.9%)	(0.0%)	(2.6%)	(2.0%)

*p* value^2^	0.929		-				0.406	

^1^Fisher's exact test; ^2^chi-squared test for homogeneity.

**Table 6:** Prevalence of a second canal in the mesiobuccal root (MB2) according to sex, age group, and dental arch of the patient evaluated.

	Sex		Age group				Arch	
	Male (*n* = 438)	Female (*n* = 562)	Children (*n* = 28)	Adults (*n* = 845)	Middle-aged (*n* = 114)	Older adults (*n* = 13)	Right (*n* = 505)	Left (*n* = 495)
MB2	311	373	21	572	81	10	332	352
	(71.0%)	(66.4%)	(75.0%)	(67.7%)	(71.1%)	(76.9%)	(65.7%)	(71.1%)

*p* value^1^	0.157		0.191				0.078	

^1^Fisher's exact test.

## Data Availability

No data were used to support this study.
